# Crestal endoscopic approach for evaluating sinus membrane elevation technique

**DOI:** 10.1186/s40729-018-0126-6

**Published:** 2018-05-17

**Authors:** Samy Elian, Khaled Barakat

**Affiliations:** 1 Faculty of Dentistry, Royal College of Surgeons in Ireland (RCSI), Dublin, Ireland; 2 Faculty of Dental Surgery, Royal College of Physicians and Surgeons of Glasgow, Glasgow, Scotland; 3 Dentistry Department, Sohag University Hospital, Sohag, Egypt; 4 Oral and Maxillofacial Surgery Department, Faculty of Dentistry, Minya University, Minya, Egypt

**Keywords:** Maxillary sinus endoscopy, Schneiderian membrane perforation, Crestal sinus lifter, Sinus implants, Endoscopic implants, Atrophic posterior maxilla

## Abstract

**Abstract:**

Closed sinus lifting is a unique technique in being simple and less invasive (Summers, Compendium 15(6):698, 1994). However, it is blind to assess the safety of sinus lining during lifting without perforation. Previously, sinus membrane was assessed endoscopically in an invasive way. We aimed to judge clinically the sinus membrane integrity after crestal elevation by a direct simple less invasive endoscopic visual assessment through the crestal osteotomy site. To confirm undetected perforation, the sinus membrane was monitored dynamically by introducing the endoscope through a trephined opening in the lateral wall of the sinus (Nkenke et al., Int J Oral Maxillofac Implants 17(4):557–66, 2002).

**Patients:**

Twelve patients suffering atrophic posterior maxillae ranging 3–5 mm bone height below the sinus membrane were included to perform closed sinus lifting with simultaneous immediate implant placement under direct endoscopic assessment.

**Results:**

The floor was lifted without perforation in 83.33% of cases. However, it varied according to its thickness. Minor perforations occurred in two cases (16.67%). Both perforations were detected from the crestal endoscopic view while one of them was detected from the lateral endoscopic approach.

**Conclusion:**

Crestal endoscopic access gives better direct vision to the membrane than the induced opening in the lateral wall of the maxillary sinus. Moreover, it uses the same prepared osteotomy site without doing any extra procedures. Perforation depends on the thickness of sinus lining and its ability to stretch during elevation. Intact crestal sinus floor elevation can never be guaranteed under endoscopic monitoring especially with thin irregular membranes.

## Introduction

The evolution of closed sinus lift techniques since 1994 [1] was proposed as a less invasive method for management of atrophic posterior maxillae [2]. However, it is a blind technique that lacks the ability to confirm an intact sinus floor elevation without perforation and thus represented a real shortcoming [3]. Various forms of osteotome lifters were designed to guarantee safe elevation of maxillary sinus membrane [4–8], but all failed to prove a non-perforated elevation during the actual lifting procedures. Previously, the endoscope was used to test the efficiency of the closed sinus lifting to detect the presence or absence of the perforation by doing a large window on the lateral sinus wall. The technique is considered an invasive surgery where another sinus surgery is required. Meanwhile, CBCT [9, 10] represented the most commonly used technique to evaluate the thickness of the membrane, but it is not sensitive enough to detect minor perforations. Thus, minor perforations can be escaped leading to implant failure. We used the crestal osteotomy to assess endoscopically directly the sinus membrane through the crestal osteotomy site of the implant.

## Patients and methods

Twelve patients (4 males and 8 females) ranging in age from 25 to 60 years were included in the study. All patients have bone height ranging 3–5 mm below the sinus membrane. They all performed closed sinus lifting and simultaneous immediate implant insertion.

**Fig. 1:**
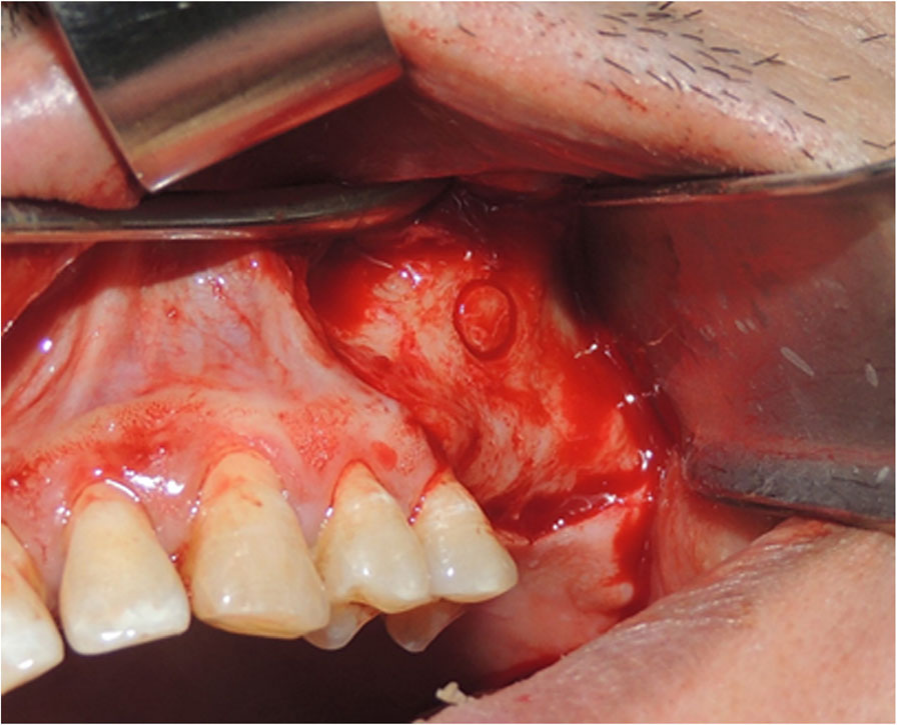
A trephined hole (4 mm bone) in the lateral wall of the maxillary sinus to allow entrance of the endoscope

Under local anesthesia, the flap was elevated and retracted exposing the crestal and buccal bone. A trephine bur 4 mm diameter on hand drill was used to make a small round window on the buccal wall of the sinus apical to the proposed implant length (Fig. 1). The trephined bony part was easily detached from the sinus membrane and placed in a bone well and covered by saline solution 0.9 ml to prevent its dryness. A rigid 1.9-mm endoscope fitted on 2.4-mm trocar with 70° lens (Karl Storz, Tuttlingen, Germany) was introduced through the prepared hole on the lateral sinus wall to visualize the actual sinus membrane lifting procedure by osteotomes and held in place by a surgeon to monitor the dynamic lifting procedure and guide the other surgeon who will use the osteotomes and place implants to achieve a safe lifting. Initial pilot drill was used to penetrate the crestal cortical bone to locate implant site.

**Fig. 2:**
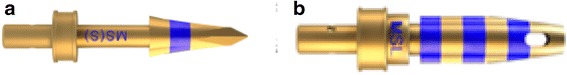
Malleting instruments supplied from InnoBioSurg (IBS) Company, Korea. **a** magic sinus splitter: used to widen and split the crest. **b** magic sinus lifter: used to lift the available bone with its attached membrane

**Fig. 3:**
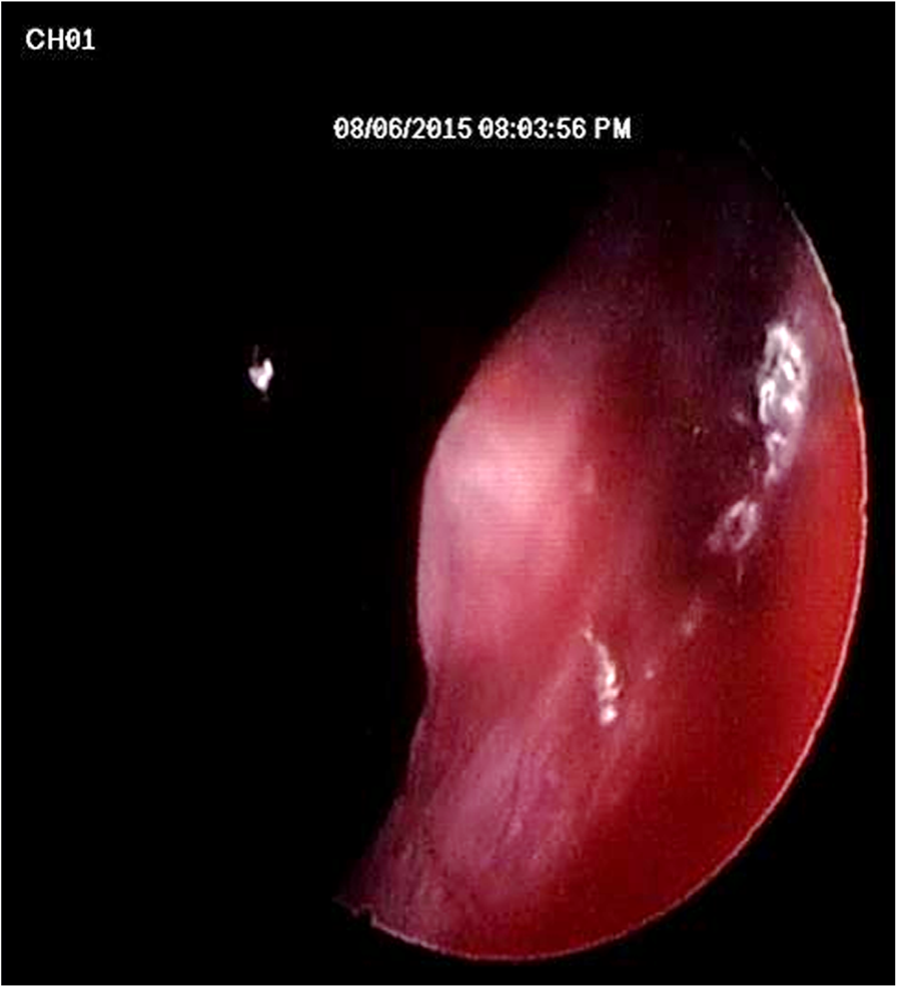
Endoscopic view from the lateral sinus wall showing the dome-shape elevation of sinus lining

Lifting technique: it consisted of two consecutive malleting instruments (Fig. 2). First was the bone splitter: a sharp, graduated arrow-like osteotome used to penetrate the maxillary bone with gentle malleting directly after using the initial drill. The splitter blades were placed on mesio-distal direction, aligned with the ridge axis and carefully malleted leaving about 1 mm of bone before reaching the sinus membrane. The second malleting instrument was the magic sinus lifter, which is a cylindrical hollow sharp-edged osteotome that was placed in a mesiodistal direction as the splitter. Under the endoscopic monitoring, the sinus lifter was gently malleted to fracture the remaining 1 mm of bone lifting it together with its attached sinus membrane toward the sinus cavity. The membrane was carefully elevated from 6 to 8 mm depending on visual assessment of the stretching capability of the membrane (Fig. 3).

**Fig. 4:**
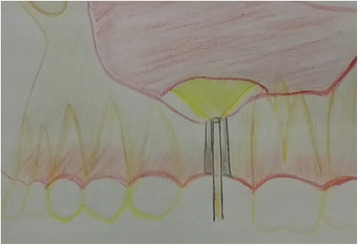
Schematic drawing showing entrance of the endoscope from the crestal osteotomy site after sinus membrane elevation to assess the integrity of the membrane

**Fig. 5:**
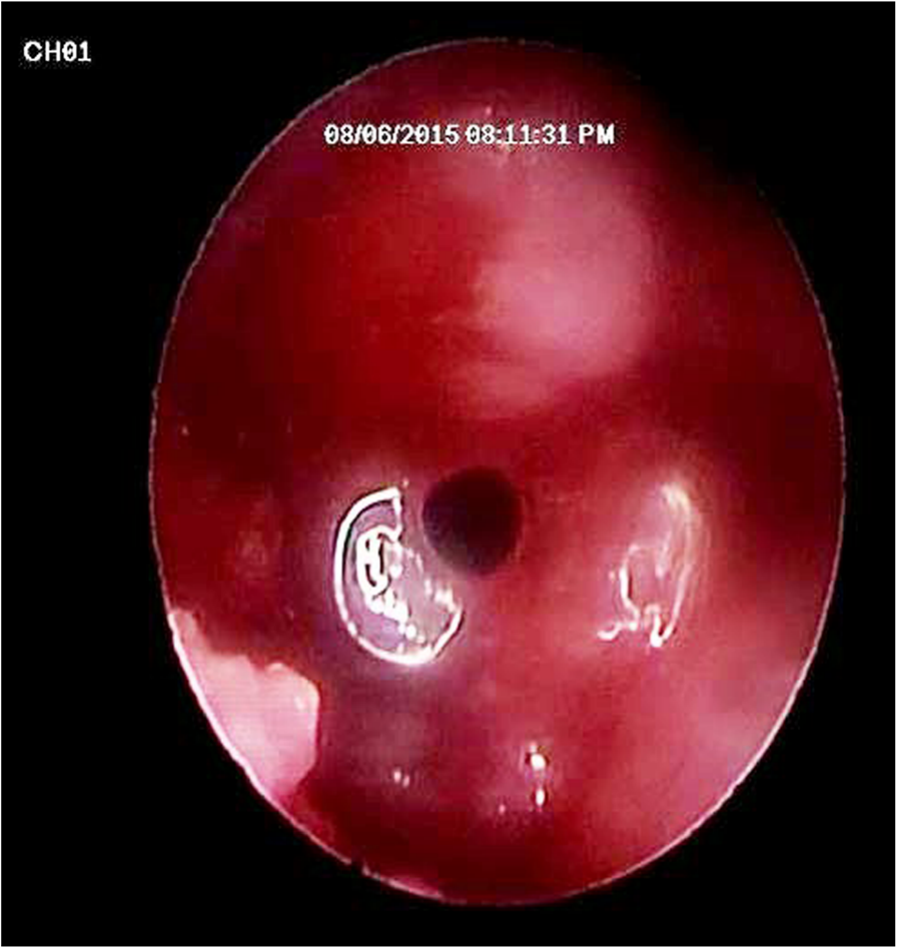
Endoscopic view from the crestal osteotomy site showing perforation of the sinus lining under the power of magnification and illumination of the endoscope

After completing the elevation of the Schneiderian membrane, the endoscope (70° lens) was removed from the lateral wall of the maxillary sinus and re-inserted (with 0°) from the crestal osteotomy site of the implant (Fig. 4) to check the integrity of the Schneiderian membrane, as well as to ensure the absence of any undetected minor perforation (Fig. 5). The implant was finally inserted in the osteotomy site to gradually lift the membrane under total endoscopic guidance from the same lateral sinus trephined hole to ensure again unperforated sinus lining from the lifting procedure during implant insertion. Afterwards, the endoscope was removed from the lateral sinus hole, and the small trephined part of bone was placed back to its original place in the buccal wall and soft tissue closed with interrupted sutures.

The sinus membrane patterns were classified into three types: flat, irregular and polyp. The membrane thickness was measured preoperatively using CBCT. The mean thickness was measured at the proposed implant osteotomy site. Patients were divided into two groups according to membrane thickness (Table 1):

- Group A (4 cases): includes membrane thickness less than 2 mm
- Group B (8 cases): includes membrane thickness more than 2 mm

**Table 1:** Descriptive statistics of membrane thickness and perforation rate

Group	Membrane thickness	Mean ± SD (mm)	Median (range)	Percentage (%) (from total)	Perforation rate (%) (from total)
A (*n* = 4)	< 2 mm	1.30 ± 0.53	1.25 (0.8–1.9)	33.33	16.67
B (*n* = 8)	> 2 mm	5.87 ± 2.70	5.50 (2.2–10.4)	66.66	0

Mucosal thickening was classified according to the criteria adopted from Soikkonen and Ainamo as follows [11]:

1. Flat: shallow thickening without well-defined outlines.
2. Semi-aspherical: thickening with well-defined outlines rising in angle of > 30° from the floor or the walls of the sinus.
3. Mucocele-like: complete opacification of the sinus.
4. Other mucosal thickening types or pathological findings.

**Fig. 6:**
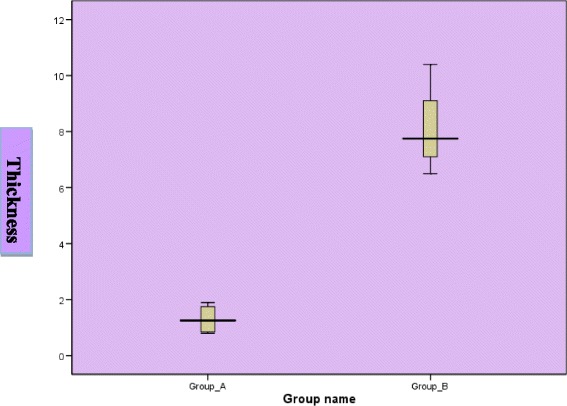
Box plot representing mean values of membrane thicknesses for the investigated groups

**Fig. 7:**
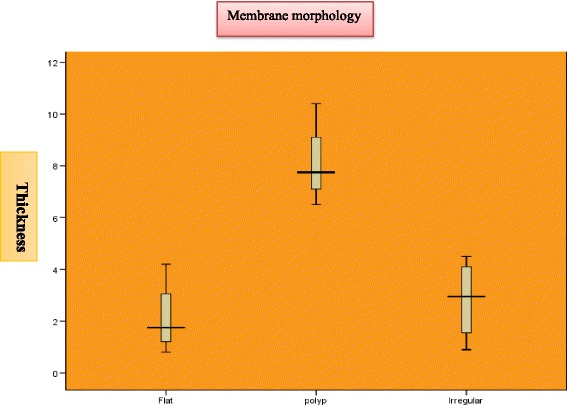
Box and Whisker plot representing median and range values of membrane thicknesses with different morphologies

Perforation occurrence was clinically monitored and recorded using the endoscopic evaluation through crestal osteotomy site. As it is a new method, we confirmed the evaluation endoscopically through a small trephined hole in the lateral sinus wall. Perforation occurrence was statistically compared to membrane thickness and type using pair-wise test, whereas Mann-Whitney *U* test was used for comparison between membrane thickness of different morphologies (Figs. 6 and 7). Chi-square was also used to show perforation rate among different groups and different morphologies.

## Results

All patients tolerated the procedure without major complications. Minor complications included postoperative swelling, edema, and pain that were managed by antibiotic and anti-inflammatory drugs. All implants were successfully osseo-integrated and loaded after about 6 months.

The floor was lifted without perforation in 83.33% of cases. The lifter was able to raise and stretch the sinus membrane safely. However, it varied according to the thickness of the membrane.

The direct observation of the sinus membrane showed that it is stretchable and can be easily elevated in eight cases where the membrane morphology was classified as thick (group B), whereas in the other four cases (group A), the membrane was thin and hardly accepted the lifting procedure (Tables 1 and 2).

**Table 2:** Chi square test showing perforation rate among different groups

Group	No perforation	Perforation	*P* value
	No. (%)	No. (%)	
Group (A)	2 (50.00)	2 (50.00)	*X*^2^ = 4.80 *P* (chi) = 0.03
Group (B)	8 (100)	0	

Percentage were expressed as row percentage

Mann-Whitney *U* test (Table 3) was used for comparison between membrane thickness and the three different morphologies. It showed a statistically significant difference between the three membrane patterns. The polyp type showed the highest statistically significantly mean membrane thickness when compared to the flat or irregular shapes, whereas the flat and irregular membranes showed no differences between their mean membrane thickness. Chi-square test (Tables 2 and 4) showed that perforation rate in different morphologies was near to significant that could be attributed to the small sample size who accepted to do a window on the lateral sinus wall.

**Table 3:** Descriptive statistics, results of Kruskal-Wallis and Mann-Whitney *U* tests for comparison between membrane thicknesses of different morphologies

Morphology	Mean ± SD (mm)	Median (range)	*P* value	Perforation rate (%)
Flat (*n* = 4)	2.12 ± 1.45	1.75 (0.8–4.20)	0.008*	0
Irregular (*n* = 4)	2.83 ± 1.64	2.95 (0.90–4.50)		16.67
Polyp (*n* = 4)	8.10 ± 1.64	7.75 (6.50–10.40)		0

*Significant at *P* ≤ 0.05

**Table 4:** Chi square test showing perforation rate by different morphologies

Morphology	No perforation	Perforation	*P* value
	No. (%)	No. (%)	
Flat (*n* = 4)	4 (100)	0	*X*^2^ = 4.80 *P* (chi) = 0.09
Irregular (*n* = 4)	2 (50.00)	2 (50.00)	
Polyp (*n* = 4)	4 (100)	0	

Percentage were expressed as row percentage

The membrane was successfully raised under direct endoscopic guidance. Regarding the elevation technique, the perforation was monitored in two cases (16.67%) under the extraordinary magnification of the endoscope. One case was early detected from the lateral approach, whereas both cases were detected from the crestal osteotomy site. Both cases were managed using PRF to seal the perforation. The implants were then immediately inserted without further complications.

There was a statistically significant relation between both groups in terms of their perforation liability, where the membrane thickness of less than 2 mm showed the highest rate of perforation (*P* = 0.008).

On the other hand, assessing the effect of membrane morphology pattern on the perforation risk revealed that the polyp type has the lowest risk of perforation, whereas the irregular type represents the most insecure pattern. There was a relation between different membrane morphology and perforation.

## Discussion

Crestal sinus lifting technique is a simple less invasive procedure. Nevertheless, it suffers a serious disadvantage of being a blind technique. Thus, perforation can easily occur without being detected which will lead to later implant failure especially when bone graft is added [1, 12–14]. We used endoscopic-assisted evaluation as a dependable method to assess the safety of the Schneiderian membrane elevation from the same crestal osteotomy site. Others used a more invasive technique by doing a window on the lateral sinus wall [15, 16].

Considering the relation between the membrane thickness and its perforation risk, our results showed a higher liability of perforation in membranes less than 2 mm thickness. Thus, we advocate that any membrane thickness less than 2 mm should not be elevated using a blind crestal osteotomy. Consequently, the membrane thickness should be precisely estimated using at least a preoperative CBCT prior to any anticipated blind elevation technique [10].

The use of lateral endoscopic approach [15, 17], despite being safe with minimal complications, can be substituted with the crestal one as in our study. The crestal endoscopic approach has some surpassed advantages. It saves the patient undue lateral bony osteotomy and membrane access perforation while using an already available access (crestal osteotomy site). An endoscope of 1.9 mm launched on 2.4 mm trocar can readily fit on the 3 mm crestal osteotomy width. Moreover, it gives direct magnification to the sinus membrane through the osteotomy site, and it is more precise in detecting almost microscopic perforations that may be even spared during lateral endoscopic examination. The raising of the sinus membrane in a closed approach proved to be a safe technique as long as there is appropriate membrane thickness more than 2 mm [5, 7]. The crestal elevation is not a technique and osteotome design dependent procedure, but it is rather a membrane structured dependent method. Endoscopic crestal evaluation represents a precise valuable and easy tool when routinely available as chair side equipment for detecting any perforations and hence modify decision making after lifting procedures.
